# Simulation Modeling to Compare High-Throughput, Low-Iteration Optimization Strategies for Metabolic Engineering

**DOI:** 10.3389/fmicb.2018.00313

**Published:** 2018-02-27

**Authors:** Stephen C. Heinsch, Siba R. Das, Michael J. Smanski

**Affiliations:** ^1^BioTechnology Institute, University of Minnesota, Twin-Cities, Saint Paul, MN, United States; ^2^Bioinformatics and Computational Biology Program, University of Minnesota, Twin-Cities, Saint Paul, MN, United States; ^3^Department of Biochemistry, Molecular Biology, and Biophysics, University of Minnesota, Twin-Cities, Saint Paul, MN, United States

**Keywords:** metabolic engineering, landscape ruggednes, numerical optimization, modeling, biosynthesis

## Abstract

Increasing the final titer of a multi-gene metabolic pathway can be viewed as a multivariate optimization problem. While numerous multivariate optimization algorithms exist, few are specifically designed to accommodate the constraints posed by genetic engineering workflows. We present a strategy for optimizing expression levels across an arbitrary number of genes that requires few design-build-test iterations. We compare the performance of several optimization algorithms on a series of simulated expression landscapes. We show that optimal experimental design parameters depend on the degree of landscape ruggedness. This work provides a theoretical framework for designing and executing numerical optimization on multi-gene systems.

## Introduction

Biotechnology applications that require the coordinated expression of dozens of genes have the potential to meet current and future needs for energy generation, production of medicinal or commodity chemicals, biosynthesis of functional biomaterials, and living biosensors (Fischbach and Voigt, 2010). Moving these complex systems between alternative host species, for example a microbial host amenable to industrial scale-up, is difficult (Galm and Shen, 2006). A major challenge is optimizing the expression levels of each required gene to maximize final output and minimize toxicity to the host cell (Lee et al., 2012; Smanski et al., 2014, 2016; Nielsen and Keasling, 2016). Technical capabilities now exist for building and testing 1000s of unique genetic constructs in parallel (Wang et al., 2009; Yuan et al., 2013; Smanski et al., 2014; Chao et al., 2017). Further, numerous improvements have been made in our ability to quantitatively control individual gene expression levels in the most commonly used organisms for industrial fermentation (Salis et al., 2009; Khalil et al., 2012; Kosuri et al., 2013; Mutalik et al., 2013; Nielsen et al., 2013; Siegl et al., 2013; Espah Borujeni et al., 2014; Bai et al., 2015; Redden and Alper, 2015; Smanski et al., 2016; Diaz de Arce et al., 2017). Leveraging both of these capabilities will enable high-throughput optimization strategies that rationally improve productivity and yield in less time than low-throughput trial-and-error approaches (Smanski et al., 2014).

**FIGURE 1:**
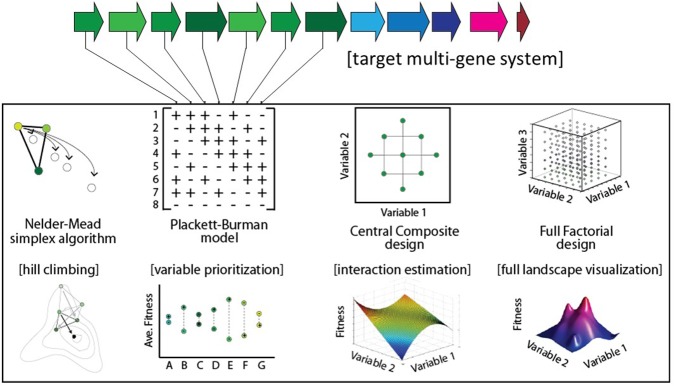
Select optimization strategies for multi-gene biological systems.

Several strategies have been proposed for genetic optimization (**Figure 1**). In the ‘multivariate modular metabolic engineering’ approach, the combinatorial design space is reduced by grouping pathway genes into operons based on previous knowledge (e.g., enzyme kinetics, branching of pathway, etc.) (Ajikumar et al., 2010; Biggs et al., 2014). The reduced combinatorial space can be elucidated empirically. For instance, this strategy was used to improve taxadiene titers ∼15,000-fold in *E. coli* (Ajikumar et al., 2010). In another example of modular multivariate optimization, Xu et al. (2013) modified the expression levels of three modules comprising nine genes involved in fatty-acid synthesis to improve fatty-acid titers 20-fold. Recently combinatorial RBS libraries designed using biophysical models (Salis et al., 2009) have been implemented in high-throughput via multiplexed automated genome engineering (Wang et al., 2009) to improve isopropanol titers 1.5-fold (Liang et al., 2017), and NADPH regeneration rates 25-fold (Salis et al., 2009; Ng et al., 2015). Alternatively, algorithmic optimization is possible using a Design of Experiments (DOE) approach. For example, the fractional factorial ‘Yates algorithm’ was used to co-optimize both gene expression and media conditions in a single experiment, resulting in an approximately fivefold improvement in 6-aminocaproic acid titer (9–48 mg/L) in *E. coli* (Zhou et al., 2015). Lastly, linear regression is an effective approach for predicting improved expression levels of a multi-gene metabolic pathway, following a small sampling of the combinatorial design space (Lee et al., 2013; Farasat et al., 2014). Previously, linear regression was shown to be capable of predicting relative titers of intermediates within engineered variants of the violacein pathway (Lee et al., 2013), and more recently regression modeling was used to increase violacein titers 3.2-fold (Xu et al., 2017).

The ability of any global search algorithm to predict optimal expression levels depends on the ruggedness of the ‘fitness landscape’ (Pitzer and Affenzeller, 2012; Lee et al., 2013). Smooth landscapes arise when variables are independent of each other and lend themselves well to linear regression approaches. However, if the landscape is rugged, with multiple local optima separated by valleys (Romero and Arnold, 2009), rational optimization methods will not be as effective (Rios and Sahinidis, 2013). Fitness landscape analyses performed on a library of nitrogen fixation gene clusters suggests that complex multi-gene systems can be moderately rugged and will not lend themselves to linear regression (Smanski, unpublished).

Numerical optimization refers to a set of techniques aimed at identifying a local or global maximum (or minimum) in a fitness landscape. A common goal for numerical optimization methods is to find the maximum with the smallest amount of computational resources, which normally correlate to the number of sampled points. For metabolic engineering, this corresponds to the number of alternative genetic designs that would have to be designed, built, and tested. In a recent comparison of numerical optimization algorithms, variations of the DIRECT search algorithm performed well (Rios and Sahinidis, 2013). The DIRECT method balances local and global searching strategies. It was designed specifically with engineering optimization in mind, where time or resource costs associated with running experiments calls for methods with efficient use of function evaluations (Jones, 2001). Unfortunately, methods that seek to optimize the efficiency of function evaluations do not distinguish between the number of iterations and the number of function evaluations per iteration. This distinction is important for genetic engineering projects. Increasing the throughput of a single design-build-test cycle can typically be done at a small fraction of the cost compared to increasing the number of design-build-test cycle iterations.

Here, we describe and model an approach to genetic optimization that combines (i) the quantification of fitness landscape ruggedness with (ii) a high-throughput, low-iteration optimization algorithm for improving genetic design. We show that the optimization parameters should be tailored for each system based on fitness landscape ruggedness. Finally, we compare the performance of this approach to several alternative hill-climbing algorithms.

## Materials and Methods

### Creation of Model Multivariate Landscapes

**FIGURE 2:**
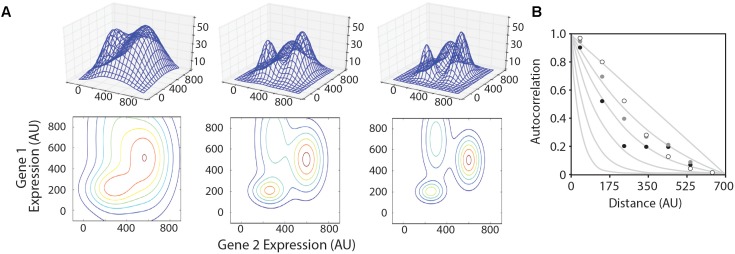
Model landscapes and ruggedness analysis. **(A)** Three model landscapes described in the text as ‘smooth’ (left), ‘medium’ (center), and ‘rugged’ (right) are shown as three-dimensional wire surfaces (top) and two-dimensional contour maps (bottom). X- and Y-axes represent hypothetical expression levels of two genes in a multi-gene system, and Z-axis represents system performance. **(B)** Autocorrelation function plotted for smooth (white circles), medium (gray circles), and rugged (black circles) landscapes compared to hypothetical traces based on NK-model, where *N* = 700 and K = 0, 1, 2, 4, 8, and 16 (gray lines, from right to left).

We created three model multivariate landscapes on which to test the optimization algorithms in this study. The landscapes were made by summing multiple three-dimensional Gaussian surfaces, the equations for which are given in Supplementary Files ‘surface_matrix-low.py,’ ‘surface_matrix-med.py,’ and ‘surface_matrix-high.py’ for the smooth, medium, and rugged landscapes, respectively. Each model landscape was designed with different levels of ruggedness by varying the X- and Y-dimensional spread of each sub-peak. The height and location in the X–Y coordinate plane of each sub-peak were maintained in each model landscape. Three-dimensional graphics of each landscape are shown in **Figure 2**.

### Quantification of Model Landscapes

Forty thousand coordinate (X,Y) points were sampled from each model landscape in a square-grid pattern (200 × 200 points) and evaluated to determine the Z-value at each location. For all possible pairwise combination of points, two values were recorded: (i) the Euclidian distance between the pairs of points in the X–Y plane, and (ii) the squared difference between the two Z-values. Next, all pairwise comparisons were binned based on Euclidian distance into bins from 0–100, 100–200, …600–700. The average variance for each bin was calculated by taking the mean of the squared differences for pairs of points in that bin. For the landscape autocorrelation analysis (LAA), we plot:

$$\mathrm{LA}=(1-\frac{\sigma_{d=\mathrm{bin}(x)}^{2}}{\sigma_{\mathrm{landscape}}^{2}})$$

where σ^2^landscape is the random variance for the landscape. This was approximated using the pairs of points for which the Euclidian distance is between 600 and 700, as distances greater than 700 are constrained by the size of the search space (1000 × 1000 grid), leading to less pairs sampled at greater distances. Landscape ruggedness was quantified by plotting lines from the function:

$$f(x)=(1-\frac{x}{N}){\left(1-\frac{k}{N}\right)}^{x}$$

for N = 700 and determining the best-fit value of k by the non-linear least squares method in R (Version 3.3.3, R Core Team, 2017).

### Simulation Algorithm for Optimizing on Model Landscapes

A series of python scripts were created to sample a quasi-random distribution of points around a defined starting coordinate, evaluate the fitness (*Z*-value) for each sampled point, and determine the center point for the next round of sampling, and iterate this process. These are included as Supplementary Files ‘SobolHillClimb.py,’ ‘SobolHillClimbWithProjection.py,’ and ‘SobolHillClimb-CMA-ES.py.’ For each algorithm, parameters that must be specified include the starting coordinates, the Sobol range radius (a measure for how broad of an area is sampled with each iteration), the number of dimensions, the number of designs to evaluate per iteration, and the fraction of top-performing designs to use in calculating the center point for the subsequent iteration. The three algorithms differ in how each iteration of sampled points is generated. In the most basic algorithm, the center point is the geometric center of top-performing designs. In the ‘projection’ algorithm, the new center point is projected twofold along a vector connecting the previous center point and the center of the top-performing designs. In the CMA-ES strategy (Hansen and Hansen, 2006), the center point is generated as described for the basic algorithm, but the subsequent quasi-random sampling is perturbed to preference sampling in the same direction as the vector connecting the previous center point to the next center point.

## Results

### Assessing the Ruggedness of a Multivariate Expression Landscape

We began by creating three model landscapes for testing optimization algorithms (**Figure 2A**). The 3D landscapes simulate a two-gene system, where the X- and Y-dimensions represent the expression levels of the two genes, and the Z-dimension represents the measured performance of the system (e.g., the product titer for a metabolic system). Most metabolic pathways are more complex than this, but we chose to model a two-gene system because the progress and results of the algorithm are easily visualized. The algorithms described in this study can be easily adapted to higher-dimensional space.

We first aimed to establish a metric for determining the ruggedness of a gene expression landscape based on Kauffman’s N–K method (Weinberger, 1991; Kaufmann, 1993). In the N–K method, N refers to the number of component parts and K is the order of interaction. When K = 0, the system variables behave independently, and the landscape is expected to be smooth. The maximal value of K is N-1, which would represent a system where the optimal level of any variable depends on the setting of all other variables. This would produce a rugged landscape. A LAA allows one to estimate the average ruggedness of a landscape using sampled data points (Weinberger, 1990; Fontana et al., 1993). LAAs have been performed in biology to problems of RNA folding and protein structure/function, but not to multi-gene expression analyses. A key difference in these types of problems is that the permutable variables in macromolecular optimization problems are discrete, whereas gene expression level is a continuous variable. We have slightly modified previous LAAs to account for this difference. For each model landscape, we sampled 40,000 points in the X,Y coordinate space to evaluate f(x,y). The autocorrelation compares the average *variance for pairs of data points* within a given Euclidian distance on the (X,Y) plane to the average variance for the landscape as a whole. On smooth landscapes, the variance of f(x,y) for two points located near each other in the (x,y) plane is expected to be small. The variance will approach the average landscape variance as distance between two points increases. The plotted landscape autocorrelation, $\left(1-\frac{\sigma^{2}d=\mathrm{bin}(x)}{\sigma^{2}\mathrm{landscape}}\right)$, is approximately 1 for very close points and approaches 0 as the distance between compared datapoints increases. The rate at which this landscape autocorrelation value decreases is related to landscape ruggedness, with more rugged landscapes dropping off more rapidly (**Figure 2B**). We quantify landscape ruggedness by comparing landscape autocorrelation plots to the equation: $f(x)=(1-\frac{x}{N}){\left(1-\frac{k}{N}\right)}^{x}$ and solving for k. The model smooth, medium, and rugged landscapes generated for testing optimization algorithms have k values of 0.832, 1.07, and 2.07, respectively. For empirical optimization of metabolic pathways, we envision that the actual landscape ruggedness would be measured with a seed library of diverse expression cassettes. Our model landscapes are in the same range of ruggedness as seen in multigene metabolic pathways for which pathway productivity is measured under combinatorial expression levels (Ajikumar et al., 2010; Lee et al., 2013; Smanski et al., 2014).

### High-Throughput, Low-Iteration Optimization Algorithms

We next developed a set of numerical optimization algorithms that are designed with the technical aspects of metabolic engineering in mind. Namely, the algorithms search the multivariate expression space with very large sampling libraries, but low numbers of iterations. As a comparison, a 20-gene synthetic nitrogen fixation pathway was recently improved using five iterations, each with approximately 100 alternative genetic designs.

**FIGURE 3:**
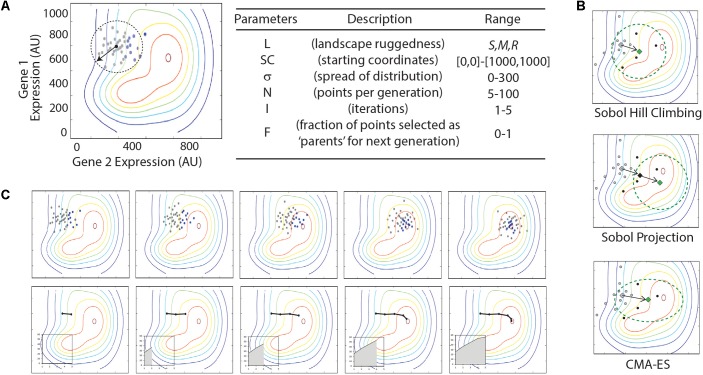
Illustration of optimization algorithms used in this study. **(A)** Illustration and table of parameters in Sobol Hill Climbing algorithm. The vector and dashed circle denote the spread of sample points (*σ*) from the starting coordinates (*SC*). Gray and black dots show the sampled points per iteration (*N*). Black dots represent the fraction of points selected as parents for the next generation (*F*). Parameters are listed in the table with approximate ranges of parameter values explored in the current study. **(B)** Top panels show results from a single simulation experiment with the following parameter values: *L = smooth; SC* = [300,700]; *σ* = x; *N* = xx; *I* = 5; *F* = 0.x. Each iteration is shown from left to right. Bottom panel shows the route taken by the optimization algorithm, with black line tracing location of center-points for Sobol sampling. Insets show increase of fitness (z-axis) through each iteration. **(C)** Summaries of triplicate simulations on three distinct parameter sets. Graphs represent optimization routes as described in **(B)**, with triplicate simulations represented as black, red, and blue lines. Parameters are given below each graph in the format of [*L; SC; σ; N; I; F*].

Each optimization algorithm follows a similar order of operations. An initial set of (x,y) coordinate points are sampled and their fitness is evaluated using the landscape function, f(x,y). The subset of points with the greatest fitness (i.e., the ‘parents’) are used to determine the center point and shape of the next set of samples (**Figure 3**). The algorithm parameters are listed in **Figure 3** and include the number of samples taken in each generation, the area of the multidimensional expression space sampled, and the fraction of sampled points carried forward as parents for the next iteration. In each case, we sample a defined area using Sobol sequences. Sobol sequences provide a quasi-random distribution of a search space and provide more even coverage of the space than a random Gaussian sampling.

Three unique optimization algorithms were tested that differ in how the new sampling space is determined for each iteration (**Figure 3**). The most simple method, which we call ‘Sobol Hill Climbing,’ takes the geometric center of the high-fitness parent points in the (x,y) plane and uses that as the center point for the next iteration of Sobol sampling (**Figure 3A**). The ‘Sobol Projection’ algorithm draws a vector from the center of the sampled space through the geometric center of the high-fitness parent points. If the distance [in the (x,y) plane] between those two points is d, the center of the next generation of sampled points is along that vector 2×d away from the previous center (**Figure 3B**). The Sobol Projection algorithm has the advantage of moving faster in an uphill direction with each generation, but it will also over-shoot the global maximum more easily than the Sobol Hill Climbing algorithm. The last and most complex algorithm uses the covariance matrix adaptation evolution strategy (CMA-ES; **Figure 3B**) (Hansen and Hansen, 2006). This algorithm differs from Sobol Hill Climbing in two important ways. First, the center point for the next iteration is determined by the weighted average of the high-fitness parent points, with weights determined by fitness value. Second, the shape of the sampling space is adjusted with each iteration. While the first two algorithms always search with a Sobol sequence following an N-dimensional standard normal distribution, the CMA-ES algorithm adjusts both the size and shape of the sampled area, according to the size and shape of the distribution of high-fitness parent points.

We evaluate the performance of an algorithm by tracking the fitness of the center point for each of the first five iterations (**Figure 3C**). The area under this curve represents the performance of the algorithm. In this way, the performance reflects both the fitness value attained and how quickly the algorithm arrived at that fitness value. We run each algorithm five times with identical parameters and record the standard deviation of the performance metric. This gives a measure for how reliably the algorithm can be expected to perform.

### Parameter Optimization for Each Algorithm

Parameters such as number of points sampled per iteration or the number of iterations are likely to be determined by the time and resources available for expression optimization efforts. Parameters affecting the distribution of sampled points and the fraction of sampled points used as parents for the next iteration do not change the cost of a given design-build-test iteration, but can greatly influence the optimization results. We simulated each optimization algorithm using a range of parameter values for σ and F. For each combination of parameters, we simulated five optimizations and score both the average fitness and the standard deviation, as measures of performance and reliability, respectively.

**FIGURE 4:**
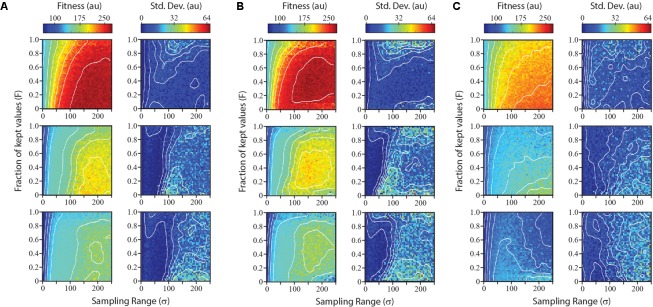
Performance and reliability of numerical optimization algorithms across parameter space. The Sobol hill climbing algorithm **(A)**, Sobol projection algorithm **(B)**, and CMA-ES algorithm **(C)** are compared. For each algorithm, left plots show mean performance from five independent simulations at each parameter combination for optimizations run on a smooth landscape (top), medium landscape (middle), and rugged landscape (bottom). Right plots show reliability of algorithm for each parameter combination, measured as the standard deviation of performance over the five independent simulations.

Results from the survey of parameter combinations for the three search algorithms are shown in **Figure 4**. Not surprisingly, each algorithm performed best on the smoothest landscape, both in terms of the gain in fitness and in the reliability. The Sobol Hill Climbing algorithm (**Figure 4A**) generally worked best when each iteration sampled a disperse set of points (large σ value) and only a small fraction of sampled points (small *F*-value) were used to seed the next generation. For medium and rugged landscapes, the algorithm was less reliable at values of *F* < 0.2. This was not observed for the smoothest landscape.

The Sobol Projection algorithm (**Figure 4B**) performed slightly better that the Sobol Hill Climbing method, particularly on more rugged landscapes. Notably, this algorithm was more sensitive to the fraction of kept values (F). Low *F*-values resulted in a substantial decrease in fitness as well as an increase in noise. Both the Sobol Projection and Sobol Hill Climbing algorithms showed a prominent loss of reliability (high standard deviation) on the medium-ruggedness landscape when the sampling range was approximately 100 units, even at intermediate *F*-values. At these parameter values, the optimization algorithm tended to get trapped in one local optimum, which was determined stochastically at an early iteration.

The CMA-ES optimization strategy (**Figure 4C**) performed substantially worse than the others in the conditions tested, both in terms of fitness values attained and in the reliability. It routinely found the global maximum in the smoothest landscape, but not as quickly as the other two algorithms. For the medium and rugged landscape, it rarely found the global maximum in the first five iterations. When the CMA-ES algorithm was allowed to run for more iterations, it routinely found the global maximum (data not shown).

## Discussion

The topology of landscapes connecting sequence space to biological phenotypes impacts the evolution of biological systems (Kaufmann, 1993). This has been shown through a combination of theoretical and experimental work, but primarily at the level of single proteins or RNA molecules (Fontana et al., 1993; Perelson and Macken, 1995). Smooth landscapes occur when the variables behave independently. Systems with smooth Mt. Fuji-like landscapes lend themselves to simple optimization approaches (Romero and Arnold, 2009). In a system comprising perfectly independent variables, each variable could be optimized separately and the optimum of each variable combined to locate the global maximum. However, in rugged or partially rugged landscapes, interactions among variables can create several local maxima or minima that will confuse optimization efforts (Romero and Arnold, 2009). In a recent comparison, problem dimensionality and non-smoothness decreased the performance of all optimization algorithms (Rios and Sahinidis, 2013) tested.

Modern DNA synthesis and assembly capabilities allow for the design, construction, and evaluation of large libraries of multi-gene systems (Smanski et al., 2014; Freestone and Zhao, 2015; Zhou et al., 2015). This enables evolution-landscape analyses that connect expression levels over each gene in the system with overall system performance. The ruggedness of multi-gene expression landscapes has never been rigorously analyzed, but is important for the performance of optimization algorithms. Linear regression optimizations require that the landscape is smooth and devoid of sub-optima (Lee et al., 2013). However, we have observed moderate ruggedness in the multivariate expression landscape of the nitrogen fixation gene cluster (Smanski et al., 2014). Landscape ruggedness in multi-gene systems can arise from several scenarios. It is possible that the landscape is rugged because of interactions between the final protein products. For example, for multi-protein complexes, optimal system performance might occur at a particular stoichiometry of component parts (Smanski et al., 2014). In this case the optimal level of each component is not fixed, but depends on the expression levels of other components in the system. A second mechanism for landscape ruggedness in multi-gene systems, which can be considered an ‘apparent ruggedness’ arises from genetic context effects (Cardinale and Arkin, 2012). These genetic context effects are often unintended consequences that arise from manipulating expression levels of different genes that are in close proximity in the DNA sequence. For example, strong transcription of one gene can attenuate the expression of a neighboring, reverse-oriented transcript via several possible mechanisms (Brophy and Voigt, 2016). Apparent ruggedness caused by genetic context effects will diminish the efficacy of linear regression and other methods that assume a smooth landscape. Whether the ruggedness of a gene expression landscape comes from interactions of gene products, or genetic context effects that produce a lot of noise when sampling a multidimensional expression space, the impact on optimization strategies is similar. The global optimum on smooth landscapes can be found through conservative searches that continuously walk uphill. Rough landscapes require a less conservative approach where a fraction of the sampling resources are used to search for other local maxima.

We have presented a set of analyses that first assess landscape ruggedness and then optimize the landscape using a limited number of high-throughput iterations. We show that landscape ruggedness affects optimal parameter settings during a multigene optimization strategy. As the landscape topology is a characteristic of the system being optimized, it will not be tunable (as it was with our model landscapes). However, knowledge of the ruggedness can guide the engineer to select appropriate parameters values such as the sampling range and the fraction of sampled points used to guide the next iteration. Smooth landscapes tolerate optimization strategies that cast a broad net over the sampling space and use information from only a small number of sampled points to direct the next round of sampling. Conversely, optimization of more rugged landscapes benefits, both in terms of performance and reliability, from sampling less broadly and using information from roughly 40% of the sampled space to direct the next round of sampling. We did not assess whether the benefit of improved optimization parameters outweighs the cost of performing an initial sampling of variable space to quantify ruggedness. Such a cost/benefit analysis would be highly specific to the system being optimized.

Landscape ruggedness assessments are likely only valid in the local neighborhood of variable space. Rugged fitness landscapes can appear smooth across small search spaces, and empirically derived fitness landscapes tend to be asymmetric (DeWitt and Yoshimura, 1998). Because of this, it is important to reassess local ruggedness in optimizations that drift far from the original starting point. While not included in the models tested here, it would be useful to continuously update the ruggedness quantification with each round of sampling. This could be done using points sampled during optimization efforts and would not require any additional experimental steps.

The modeling we have performed in this study optimizes over a landscape with two independent variables (X and Y axes; representing the gene expression from two different genes), and one dependent variable (Z axis; representing system fitness). We chose a simple system for ease of visualization of how the algorithm functions to climb in three-dimensional space. Each of the components of our work flow will work equally well for any N-dimensional optimization. For example, a 10-gene metabolic pathway would contain 10 independent variables representing expression levels of each gene and an 11^th^ dependent variable corresponding to the final titer of the molecule of interest. Because we ran our simulation experiments on a relatively low-dimensional space, we decreased the number of sampled points per iteration accordingly. For an 8–12 gene metabolic pathway, an analogous experiment would require 100–200 sampled points per iteration. This scale is in line with recently demonstrated capabilities (Smanski et al., 2014).

## Conclusion

We propose an integrated strategy for metabolic pathway engineering that combines landscape analysis with a multivariate optimization algorithm. An initial autocorrelation analysis provides a quantitative measure of the ruggedness of the adaptive landscape. This ruggedness metric is used to guide an appropriate selection of parameters during the iterative optimization process. Of the three optimization strategies simulated in this study, the Sobol Projection method gave the best performance on several model landscapes. Further work is needed to validate this strategy using an experimental system.

## Author Contributions

SH, SD, and MS designed the experiments and performed the analyses. SH and MS wrote the manuscript.

## Conflict of Interest Statement

The authors declare that the research was conducted in the absence of any commercial or financial relationships that could be construed as a potential conflict of interest.
